# Retrieval of Two-Dimensional Size Distribution and Concentration of SiO_2_@Au Core–Shell Nanospheres by Extinction Spectroscopy

**DOI:** 10.3390/nano16201305

**Published:** 2026-10-16

**Authors:** Fengyi Dou, Paerhatijiang Tuersun, Guoming He, Jingxian Wang

**Affiliations:** Xinjiang Key Laboratory of Luminescence Minerals and Optical Functional Materials, School of Physics and Electronic Engineering, Xinjiang Normal University, Urumqi 830054, China; 107622024210502@stu.xjnu.edu.cn (F.D.); 107622024210501@stu.xjnu.edu.cn (G.H.); 107622024210497@stu.xjnu.edu.cn (J.W.)

**Keywords:** SiO_2_@Au core–shell nanospheres, localized surface plasmon resonance, extinction spectroscopy, particle size characterization, non-negative Tikhonov regularization

## Abstract

The optical properties of SiO_2_@Au core–shell nanospheres are related to the core radius, shell thickness, and particle number concentration. However, simultaneously retrieving their joint distribution of core radius and shell thickness, together with particle number concentration from extinction spectra, is challenging because of parameter coupling and ill-posedness. In this work, we address issues in retrieving this two-dimensional size distribution and concentration of SiO_2_@Au core–shell nanospheres from extinction spectra. A standardized inversion strategy applicable to structural configurations is established through physical mechanism verification, the optimization of inversion parameters, and robustness evaluation. The results show that the optimal inversion parameters depend on particle structure. For small-core/thin-shell nanospheres, the optimal wavelength range is 200–400 nm with a wavelength interval of 5 nm. For the three structural categories, the optimal wavelength range extends over 200–1200 nm with a wavelength interval of 10 nm. In simulated tests with random noise levels ranging from 0.01% to 1%, the relative errors in the retrieved mean core radius and shell thickness are below 2% for all systems, indicating stable recovery of the mean structural parameters under the specified noise conditions. These numerical results provide a theoretical proof of concept; experimental validation with real SiO_2_@Au nanoparticles remains necessary before practical application.

## 1. Introduction

As one of the plasmonic functional materials with the greatest potential for clinical translation in nanophotonics and biomedicine [1,2,3,4], silica-core/gold-shell composite nanoparticles (SiO_2_@Au) integrate the excellent chemical stability and biocompatibility of the SiO_2_ core with the unique localized surface plasmon resonance (LSPR) properties of the Au shell. The LSPR originates from the collective coherent oscillation of free electrons in the Au shell under incident light excitation [5,6], which can generate local electromagnetic-field enhancement by factors of 10^3^–10^6^ at the nanoscale. Moreover, the resonance wavelength can be continuously tuned over a broad spectral range from the ultraviolet to the near-infrared region, typically spanning 200–1200 nm [7]. This special optical property endows SiO_2_@Au core–shell nanoparticles with irreplaceable application value in the construction of surface-enhanced Raman scattering substrates [8,9], precision photothermal therapy of tumors [10], multimodal bioimaging [11], and highly sensitive biochemical sensing [12,13]. It should be emphasized that the ultimate performance of these applications is directly governed by nanoscale-precision regulation of the LSPR response. Nanoscale fluctuations in shell thickness can lead to order-of-magnitude variations in surface plasmon resonance sensitivity and near-field enhancement [14]. Deviation of the LSPR peak from the biological optical window of 700–900 nm can markedly reduce photothermal conversion efficiency, necessitating higher laser power and thereby increasing the risk of nonspecific tissue damage [15,16]. However, existing single-particle characterization techniques are unable to simultaneously and quantitatively resolve the two-dimensional joint distribution of core radius and shell thickness, as well as particle number concentration [17]. Therefore, methods capable of simultaneously estimating these key parameters could be valuable for evaluating structural uniformity and optimizing the performance of plasmonic nanomaterials. However, the suitability of extinction spectroscopy-based inversion for high-throughput characterization and industrial quality control depends on experimental factors such as measurement repeatability, instrument stability, sample heterogeneity, batch-to-batch variation, and computational processing time, and these aspects require further validation using real samples, repeated measurements, multiple concentrations, and independently prepared batches.

It is worth noting that Au–SiO_2_ core–shell nanostructures have been extensively studied, including Au@SiO_2_ structures with a gold core and a silica shell and SiO_2_@Au structures with a silica core and a gold shell. The current nanoparticle characterization toolbox remains insufficient to meet the demand for high-throughput and precise characterization of the two-dimensional geometric parameters and particle number concentration of Au–SiO_2_ core–shell nanoparticles. Transmission electron microscopy and scanning electron microscopy are currently reliable and widely used techniques for single-particle morphological characterization [18,19,20]. However, they suffer from three fundamental limitations. First, the limited sampling size prevents accurate representation of the overall statistical distribution within a batch [21,22,23,24]. Second, the complex and destructive sample preparation procedures can introduce artifacts such as aggregation and structural deformation [25,26,27,28]. Third, these techniques are difficult to implement for in situ, real-time monitoring of the synthesis process [29,30,31]. Dynamic light scattering, although simple to operate and low in cost, can only measure the hydrodynamic diameter and cannot effectively distinguish the core and shell information of core–shell structures. Moreover, it is highly sensitive to particle aggregation, and the measured sizes in polydisperse systems are generally overestimated [32,33,34]. Atomic force microscopy (AFM) can achieve a vertical resolution of up to 0.1 nm. However, its lateral resolution is limited, and sampling efficiency is low. In addition, contact-mode AFM may induce mechanical damage to soft nanoparticles [35,36]. In contrast, extinction spectroscopy is based on the interaction between light and discrete particle systems within the framework of Maxwell’s electromagnetic theory [37]. By quantitatively retrieving particle number concentration and size distribution from extinction spectra, this technique offers key advantages including non-destructive measurement, rapid analysis, high throughput, and in situ quantitative capability [38,39]. A single measurement requires only a few minutes and can provide ensemble-averaged statistical information from millions of particles, making it an ideal technical route for quality control in the scalable production of core–shell nanoparticles and for real-time monitoring of synthesis processes [40]. In recent years, it has become a research hotspot in the field of nano-optical characterization.

To our knowledge, a mature, reliable, and general solution has not yet been established for retrieving the two-dimensional size distribution and number concentration of SiO_2_@Au core–shell nanoparticles from extinction spectra, either in terms of the underlying physical mechanism or numerical computation. From the physical perspective, the extinction spectra of SiO_2_@Au core–shell nanoparticles are jointly modulated by the core radius and shell thickness. On the one hand, both an increase in core radius and a decrease in shell thickness can induce a redshift of the LSPR peak [41,42], indicating an intrinsic coupling effect between their influences on spectral features. If the independent modulation mechanisms of these two parameters on the extinction spectrum cannot be physically clarified, two-dimensional retrieval will suffer from severe parameter degeneracy, making it difficult to obtain a stable and unambiguous solution without additional physical constraints or regularization. On the other hand, extinction-spectrum inversion for polydisperse systems is governed by a Fredholm integral equation of the first kind, representing a typical severely ill-posed linear inverse problem. Even small experimental noise can be markedly amplified by the ill-conditioned matrix, leading to a sharp deterioration in the stability and accuracy of the retrieved results, and may even produce nonphysical solutions [43]. Random perturbations are widely used as a controlled noise model in numerical studies of ill-posed particle size distribution inversion [44]. Although methods such as non-negative Tikhonov regularization can suppress ill-posedness to some extent, the accuracy and stability of the inversion still strongly depend on the selection of key preset parameters, including the size discretization step, spectral range, and wavelength sampling interval. Our group has previously developed extinction spectroscopy-based inversion methods for retrieving the size distribution and particle number concentration of nanospheres and nanospheroids [45,46], and has demonstrated the reliability and advantages of this technique for high-throughput characterization of nanoparticles with simple geometries. However, investigations on two-dimensional inversion for core–shell structures remain limited. Moreover, existing approaches generally rely on empirically fixed parameters and do not account for differences in optical sensitivity among different core–shell configurations, resulting in limited general applicability and uncertain inversion accuracy.

In this work, SiO_2_@Au core–shell nanospheres are selected as the research object, and a framework for two-dimensional size distribution and concentration inversion is established, covering physical mechanism validation, key parameter optimization, and robustness evaluation. First, the independent modulation mechanisms of the two geometric parameters on the extinction spectrum are clarified. Subsequently, the effect of key preset parameters on the ill-posedness of the inversion is analyzed. Finally, the practical applicability of the proposed method is verified through noise tests. Accordingly, the present study focuses on the theoretical development and numerical assessment of an inversion framework for retrieving the two-dimensional size distribution and concentration of SiO_2_@Au core–shell nanospheres from extinction spectra. The spectra analyzed in this work are generated numerically using the forward model described below, and the reconstruction performance is evaluated under the specified model assumptions and simulated noise conditions. The aim is to examine the feasibility and limitations of the proposed inversion strategy; experimental validation using synthesized SiO_2_@Au nanoparticles is beyond the scope of the present study, and further assessment using experimentally measured spectra will be an important direction for future work.

## 2. Theory and Methodology

**Figure 1:**
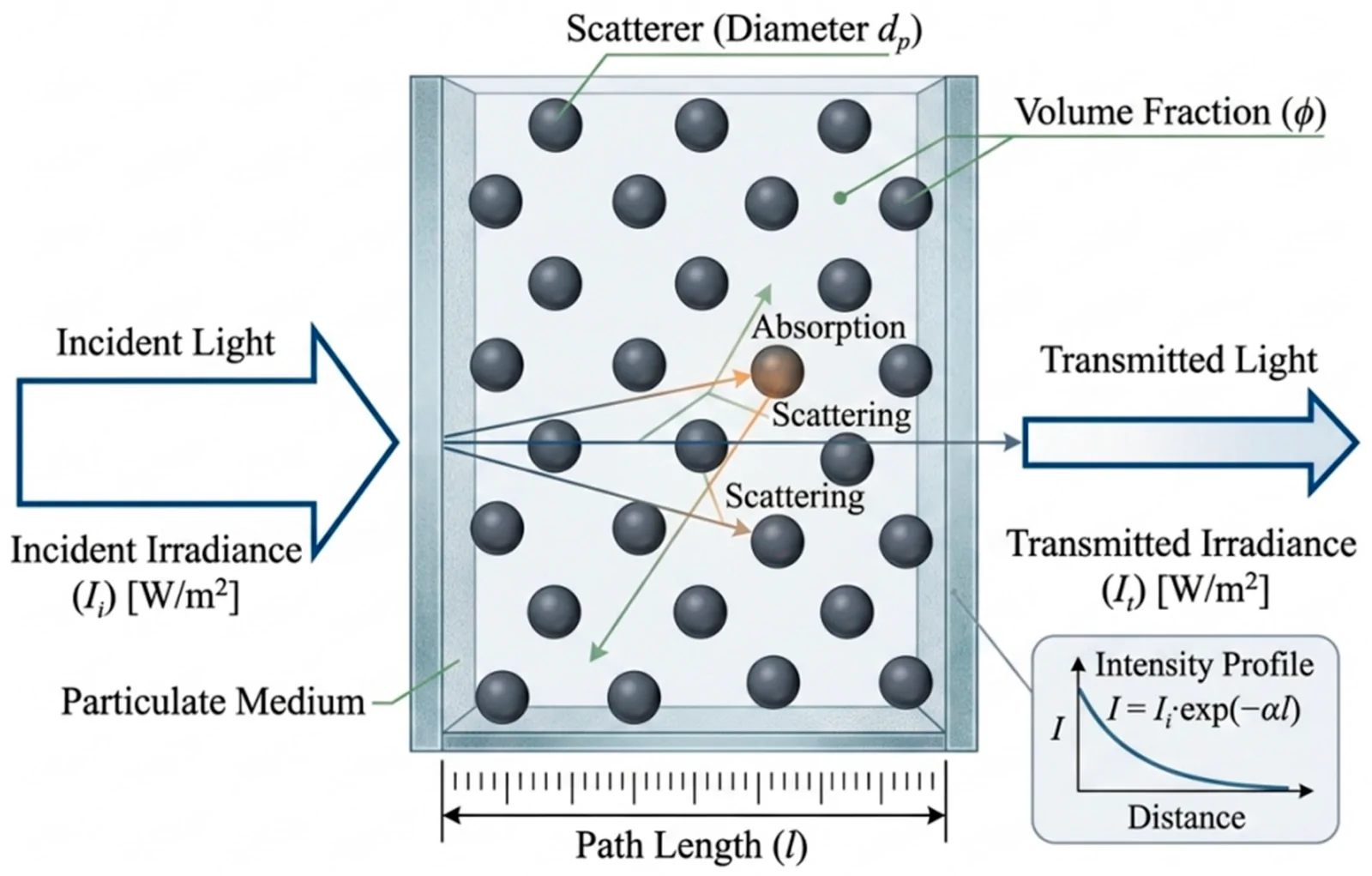
Schematic diagram of light extinction by particulate media.

The extinction spectroscopy method is a non-invasive optical characterization technique based on the Maxwell electromagnetic theory framework governing the interaction between light and discrete particle systems. By measuring the wavelength-dependent attenuation of transmitted light intensity after the incident beam passes through a particulate medium over a broad spectral range, this technique enables the quantitative retrieval of intrinsic properties of the particle system, including geometric morphology, optical parameters, number concentration, and size distribution. Extinction refers to the physical phenomenon in which the total optical power in the forward-transmission direction is attenuated due to the combined effects of intrinsic absorption and elastic scattering of the incident electromagnetic wave by the particles, as illustrated in Figure 1. Here, *I_t_* and *I_i_* denote the light intensities after and before the beam passes through the particulate medium, respectively, and *l* represents the optical path length of the beam propagating through the medium.

According to the Beer–Lambert law, the transmittance *T* is expressed as

*T* = *I_t_*/*I_i_* = exp(−*τl*) (1)

where *τ* is defined as the total extinction coefficient per unit optical path length contributed by both absorption and scattering, referred to as the turbidity of the particulate medium. In extinction spectroscopy, each particle is required to satisfy the single-scattering condition; that is, when the separation between two particles exceeds 2.5 times the particle diameter, the positional correlation becomes negligible and the light scattering can be treated as incoherent single scattering.

For a monodisperse SiO_2_@Au core–shell nanoparticle system, the turbidity arising from light scattering and absorption is expressed as

(2) $$\tau =N\cdot C_{\mathrm{ext}}\left(\lambda ,R_{c},t,n_{\mathrm{shell}},n_{\mathrm{core}},n_{m}\right)$$

where *N* is the number concentration of particles, defined as the number of particles per unit volume. The extinction cross-section *C_ext_* of a core–shell particle is a function of the wavelength of the incident light in vacuum *λ*, the core radius *R_c_*, the shell thickness *t*, the refractive index of the particle core *n*_core_, the refractive index of the particle shell *n*_shell_, and the refractive index of the surrounding medium *n*_m_, and can be solved using Mie scattering theory. In the present model, the absorbance was calculated from the extinction cross-section by assuming that the extinction contributions of individual particles can be linearly superposed. This independent-particle treatment corresponds to the single-scattering approximation and requires a sufficiently dilute and homogeneous suspension. In this study, the particle number concentration was fixed at 1 × 10^10^ cm^−3^. Therefore, the single-scattering approximation was applied specifically to this concentration under the optical path length, wavelength range, and particle size distributions considered in the present work. For a monodisperse SiO_2_@Au core–shell nanoparticle system, the absorbance *A* reflects the degree of attenuation of the light beam as it propagates through the particulate medium, providing a theoretical basis for the retrieval of particle size and concentration. According to the definition of absorbance *A* for a monodisperse core–shell particle system, it can be expressed as

*A* = log_10_(*I_i_*/*I_t_*) (3)

Substituting Equations (1) and (2) into Equation (3), the absorbance of the monodisperse SiO_2_@Au core–shell nanoparticle system can be expressed as

(4) $$A=\frac{\mathrm{lN}}{\ln(10)}\cdot C_{\mathrm{ext}}\left(\lambda ,R_{c},t,n_{\mathrm{shell}},n_{\mathrm{core}},n_{m}\right)$$

In practice, however, ideal monodisperse particle systems rarely exist. Instead, polydisperse particle systems are commonly approximated as equivalent monodisperse systems. For a polydisperse SiO_2_@Au core–shell nanoparticle system, the particle size is not uniform but follows a certain size distribution. For each particle in the polydisperse SiO_2_@Au core–shell nanoparticle system to satisfy the single-scattering condition, the turbidity of a polydisperse particle system with a number concentration of *N* arising from light scattering and absorption is expressed as

(5) $$\tau =N\sum_{j=1}^{J}\sum_{i=1}^{I}p\left(R_{c,j},t_{i}\right)\cdot C_{\mathrm{ext}}\left(\lambda ,R_{c,j},t_{i},n_{\mathrm{shell}}(\lambda ),n_{\mathrm{core}},n_{m}(\lambda )\right)$$

where *p* (*R_c,j_*, *t_i_*) denotes the particle size distribution function of the polydisperse SiO_2_@Au core–shell nanoparticle system, which must satisfy the standard normalization condition

(6) $$\sum_{j=1}^{J}\sum_{i=1}^{I}p\left(R_{c,j},t_{i}\right)=1$$

Accordingly, when the polydisperse SiO_2_@Au core–shell nanoparticle system satisfies the single-scattering condition, the absorbance *A* can be expressed as

(7) $$A=\frac{\mathrm{lN}}{\ln(10)}\sum_{j=1}^{J}\sum_{i=1}^{I}p\left(R_{c,j},t_{i}\right)\cdot C_{\mathrm{ext}}\left(\lambda ,R_{c,j},t_{i},n_{\mathrm{shell}}(\lambda ),n_{\mathrm{core}},n_{m}(\lambda )\right)$$

The particle size distribution and number concentration retrievable from the absorbance *A* of the polydisperse SiO_2_@Au core–shell nanoparticle system satisfy the following linear equation:

**A** = **CP** (8)

The expressions for matrices **C**, **P** and **A** are given respectively as

(9) $$C=\frac{l}{\ln(10)}\left[C_{R_{c_{1}}}\;C_{R_{c_{2}}}\cdots C_{R_{c_{J}}}\right],$$

(10) $$C_{R_{c_{j}}}=\left[\begin{array}{cccc} C_{\mathrm{ext}}(\lambda_{1},R_{c_{j}},t_{1}) & C_{\mathrm{ext}}(\lambda_{1},R_{c_{j}},t_{2}) & \cdots & C_{\mathrm{ext}}(\lambda_{1},R_{c_{j}},t_{I})\\ C_{\mathrm{ext}}(\lambda_{2},R_{c_{j}},t_{1}) & C_{\mathrm{ext}}(\lambda_{2},R_{c_{j}},t_{2}) & \cdots & C_{\mathrm{ext}}(\lambda_{2},R_{c_{j}},t_{I})\\ \vdots & \vdots & \ddots & \vdots \\ C_{\mathrm{ext}}(\lambda_{K},R_{c_{j}},t_{1}) & C_{\mathrm{ext}}(\lambda_{K},R_{c_{j}},t_{2}) & \cdots & C_{\mathrm{ext}}(\lambda_{K},R_{c_{j}},t_{I}) \end{array}\right],\;j=1,2,\ldots ,J$$

(11) $$P=N\cdot {\left[p(R_{c1},t_{1}),p(R_{c1},t_{2}),\ldots ,p(R_{c1},t_{I}),p(R_{c2},t_{1}),\ldots ,p(R_{\mathrm{cJ}},t_{I})\right]}^{T}$$

(12) $$A={\left[A(\lambda_{1}),A(\lambda_{2}),\ldots ,A(\lambda_{K})\right]}^{T}$$

where the superscript *T* denotes the transpose of a vector; *K*, *J*, and *I* represent the number of discrete points corresponding to the incident wavelength, core radius, and shell thickness, respectively. **C** is a *K* × (*J* × *I*) matrix representing the collection of extinction cross-sections associated with different size–shell thickness combinations across all wavelength channels, computed via the coated-sphere Mie scattering theory. **P** is a (*J* × *I*) × 1 column vector constituting the unknown to be solved, which encodes the number concentration distribution of particles corresponding to each size–shell thickness combination. **A** is a *K* × 1 column vector representing the absorbance measured at different wavelengths.

Equations (7)–(12) define the forward mapping from the two-dimensional structural distribution to the measured absorbance spectrum. For a given combination of core radius *R_c,j_* and shell thickness *t_i_*, the Mie model determines a wavelength-dependent extinction response *C_ext_*(*λ*, *R_c,j_*, *t_i_*). The absorbance of the polydisperse system is then obtained by summing the responses of all discrete core radius/shell thickness combinations according to their number concentration weights. In the matrix representation, each column of **C** corresponds to the extinction response of one specific structural combination, whereas **P** contains the corresponding number concentrations. Thus, the forward problem maps a two-dimensional structural distribution P to the absorbance vector **A** through **A** = **CP**. In this work, practical identifiability refers to the ability to numerically distinguish different core radius/shell thickness combinations from their absorbance spectra within the tested structural ranges, wavelength ranges, wavelength intervals, discretization settings, regularization conditions, and noise levels. This definition does not imply global mathematical uniqueness of the inverse problem.

**Figure 2:**
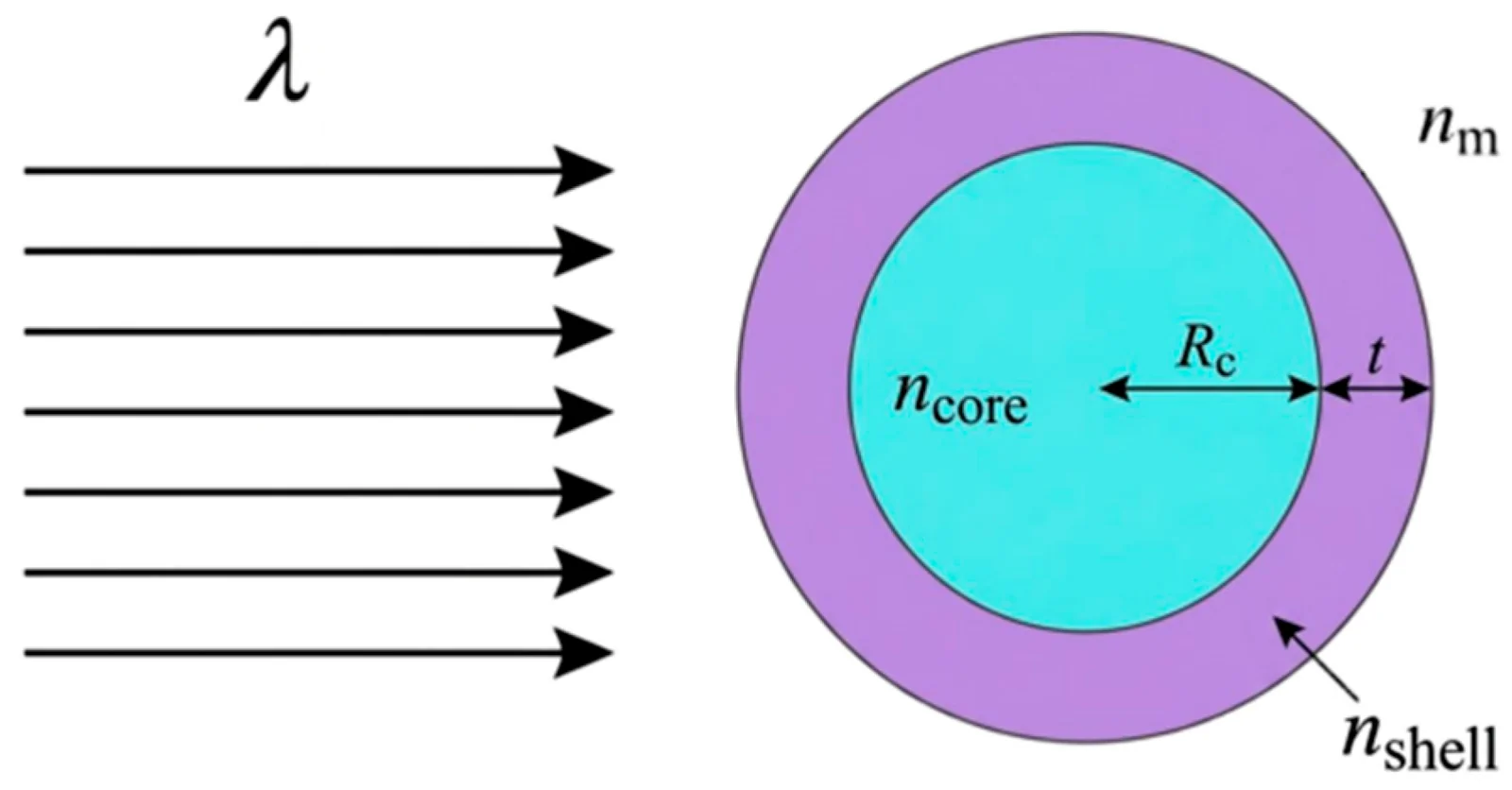
Schematic diagram of the interaction between incident light and nanoparticles.

Regarding the extinction cross-section of SiO_2_@Au core–shell nanoparticles, Mie theory provides a rigorous theoretical framework for characterizing the optical properties of coated spherical nanoparticles. In the forward extinction model, the optical properties of the SiO_2_@Au core–shell nanoparticles and the surrounding medium were treated as wavelength-dependent inputs. The complex refractive index of the Au shell was calculated using the modified Lorentz–Drude model [47] implemented in the present study, including the size-dependent damping correction. The refractive index of the SiO_2_ core was calculated using the Sellmeier dispersion relation for fused silica reported by Malitson [48], while the refractive index of the surrounding medium was calculated using the wavelength-dependent dispersion relation for water reported by Daimon and Masumura [49]. These optical inputs were treated as fixed parameters in the baseline extinction calculation and inversion. As illustrated in Figure 2, *λ* denotes the incident wavelength, *R_c_* the core radius, and *t* the shell thickness. Here, *m*_1_ = *n*_core_/*n*_m_ is the refractive index of the core relative to the surrounding medium, *m*_2_ = *n*_shell_/*n*_m_ is the refractive index of the shell relative to the medium, and *n*_m_ denotes the refractive index of the surrounding medium. Accordingly, the extinction cross-section *C_ext_* of a coated spherical nanoparticle can be expressed as

(13) Cext=2πk2∑n=1∞(2n+1)·Rean+bn

where *k* = 2π*n*_m_/*λ* is the wavenumber, *a_n_* is the electric-field component scattering coefficient, *b_n_* is the magnetic-field component scattering coefficient, and *n* is the order of the scattering coefficients.

To further evaluate the ability of the extinction spectrum to distinguish the effects of these two structural parameters, the local spectral sensitivities to the core radius and shell thickness are introduced:

(14) $$S_{R_{c}}(\lambda ;R_{c},t)=\frac{\partial C_{\mathrm{ext}}(\lambda ,R_{c},t,\ldots )}{\partial R_{c}}$$

(15) $$S_{t}(\lambda ;R_{c},t)=\frac{\partial C_{\mathrm{ext}}(\lambda ,R_{c},t,\ldots )}{\partial t}$$

*S_R__c_* quantifies the local change in the extinction cross-section caused by a unit change in the core radius with the shell thickness fixed, whereas *S_t_* quantifies the corresponding change caused by a unit change in the shell thickness with the core radius fixed. Because the extinction response is calculated numerically using the Mie model, the two derivatives are evaluated using central finite differences:

(16) $$S_{R_{c}}(\lambda ;R_{c},t)\approx \frac{C_{\mathrm{ext}}(\lambda ,R_{c}+\Delta R_{c},t,\ldots )-C_{\mathrm{ext}}(\lambda ,R_{c}-\Delta R_{c},t,\ldots )}{2\Delta R_{c}}$$

(17) $$S_{t}(\lambda ;R_{c},t)\approx \frac{C_{\mathrm{ext}}(\lambda ,R_{c},t+\Delta t,\ldots )-C_{\mathrm{ext}}(\lambda ,R_{c},t-\Delta t,\ldots )}{2\Delta t}$$

Δ*R_c_* and Δ*t* denote the perturbation steps applied to the core radius and shell thickness, respectively. The perturbation steps are selected consistently with the corresponding discretization intervals used in the inversion, while all other structural and optical parameters are kept unchanged during each perturbation. A large absolute sensitivity indicates that the corresponding parameter produces a relatively large local change in the extinction spectrum at the given wavelength. However, the sensitivity magnitude alone cannot determine whether the two parameters are distinguishable because different parameters may produce similar wavelength-dependent response patterns. Therefore, the sensitivity values evaluated at the K selected wavelengths are arranged into two vectors

(18) $$S_{R_{c}}={\left[S_{R_{c}}(\lambda_{1}),S_{R_{c}}(\lambda_{2}),\ldots ,S_{R_{c}}(\lambda_{K})\right]}^{T}$$

(19) $$S_{t}={\left[S_{t}(\lambda_{1}),S_{t}(\lambda_{2}),\ldots ,S_{t}(\lambda_{K})\right]}^{T}$$

The local spectral coupling between the core radius and shell thickness is quantified using the cosine similarity of the two sensitivity vectors:

(20) $$|\rho_{R_{c}t}|=\frac{\left|S_{R_{c}}^{T}S_{t}\right|}{{\left\|S_{R_{c}}\right\|}_{2}{\left\|S_{t}\right\|}_{2}}$$

The coefficient |*ρR*_c_*t|* is the cosine similarity between the two sensitivity vectors and is used to quantify their local spectral coupling. A value of |*ρR*_c_*t|* close to one indicates similar wavelength-dependent response patterns and stronger local parameter coupling, whereas a smaller value indicates greater local spectral separability. This coefficient is a local measure of spectral-response similarity rather than a rigorous criterion for global uniqueness. Its value depends on the selected structural point, wavelength range, wavelength sampling, and perturbation steps. The sensitivity and coupling analysis also provides a physical interpretation of the ill-posedness of the inverse problem. When neighboring core radius/shell thickness combinations produce similar extinction-response vectors, the corresponding columns of the response matrix **C** become highly correlated.

Mathematically, the retrieval of the joint size–shell thickness distribution and concentration of a polydisperse SiO_2_@Au core–shell nanoparticle system constitutes an ill-posed linear inverse problem. Neighboring combinations of core radius and shell thickness may produce similar extinction responses, resulting in correlated columns in the response matrix **C**. Consequently, small perturbations in the measured absorbance may cause substantial changes in the retrieved distribution when conventional least-squares inversion is used. The non-negative Tikhonov regularization described below is therefore introduced to stabilize the numerical inversion and to constrain the solution to physically meaningful concentrations.

(21) $$J(P)=\|\mathrm{CP}-A{\|}_{2}^{2}+\gamma \|\mathrm{LP}{\|}_{2}^{2}$$

where the fitting residual term $\|\mathrm{CP}-A{\|}_{2}^{2}$ measures the difference between the inverted spectrum and the measured spectrum, ensuring the data consistency and accuracy of the inversion results. The Tikhonov regularization penalty term $\gamma \|\mathrm{LP}{\|}_{2}^{2}$ suppresses high-frequency noise oscillations by constraining the smoothness of the solution, guaranteeing the numerical stability of the inversion results. Here, L denotes the second-order difference smoothing matrix (which exhibits optimal smoothing performance for two-dimensional particle size distributions), and *γ* > 0 is the regularization parameter that controls the weight balance between the two terms. The non-negativity constraint **P** ≥ 0, a hard constraint based on physical reality, states that the particle number concentration cannot be negative. The non-negative constraint and Tikhonov penalty improve the stability and physical plausibility of the retrieved distribution, but they do not constitute a mathematical proof of global uniqueness.

The regularization parameter *γ* was determined automatically using Morozov’s discrepancy principle. Specifically, the noise level was first estimated from the residual of the truncated-SVD solution

(22) $$\hat{\sigma }=\frac{\|b-{\mathrm{AP}}_{\mathrm{tr}}{\|}_{2}}{\sqrt{m-r}}$$

where *m* is the number of measurements and *r* is the estimated effective rank. The target residual was then defined as $\delta =\sqrt{m}\hat{\sigma }$. The value of *γ* was selected numerically such that

‖**AP***_γ_* − **b**‖_2_≈*δ* (23)

Thus, *γ* was determined from the estimated noise level rather than manually assigned. When the estimated noise level was zero or the discrepancy condition was not applicable, the SVD pseudoinverse solution was used.

To quantify the numerical conditioning of the inversion, the 2-norm spectral condition number of the inversion matrix *C* was calculated as

(24) $$\kappa_{2}(C)=\frac{\sigma_{\max}(C)}{\sigma_{\min}(C)}$$

where σ_max_(*C*) and σ_min_(*C*) denote the largest and smallest singular values of *C*, respectively. In the present implementation, *C* is constructed from the extinction responses corresponding to the inversion grids *R_c,j_* and *t_i_*. The core radius and shell thickness parameters used to define the original particle distributions were varied among the test cases. These parameters changed the calculated observation vector, *J*, but did not change the inversion matrix *C*, because the same inversion grids, optical parameters, wavelength grid, and discretization scheme were used in all cases.

To solve the non-negatively constrained Tikhonov regularization problem, the projected gradient descent method is employed. In each iteration, the updated solution is projected onto the non-negative orthant, ensuring that all iterates remain physically meaningful and satisfy the non-negativity constraint. The inversion accuracy is quantitatively evaluated using the relative error, defined as

(25) $$E(x)=\left|\frac{x_{\mathrm{inv}}-x_{\mathrm{true}}}{x_{\mathrm{true}}}\right|\times 100\%$$

where *x*_inv_ denotes the inverted value and *x*_true_ denotes the true value. To comprehensively assess the accuracy and robustness of the inversion method, five evaluation metrics are considered, including the mean core radius, the standard deviation of the core radius, the mean shell thickness, the standard deviation of the shell thickness, and the particle number concentration.

## 3. Results and Discussion

Based on extinction spectroscopy, the two-dimensional particle size distribution and concentration of SiO_2_@Au core–shell nanoparticles are characterized using the Mie scattering theory and the non-negative Tikhonov regularization method. The present inversion assumes spherical SiO_2_@Au particles with smooth, concentric shells and describes shell thickness using a single radial parameter for each particle. Therefore, particle nonsphericity, shell-surface roughness, and intraparticle shell thickness nonuniformity are not explicitly represented in the forward model. First, for monodisperse SiO_2_@Au core–shell nanoparticles, inversion is performed without adding random noise to the extinction absorbance spectrum, quantitatively verifying the one-to-one correspondence between the two-dimensional particle size and the resonance wavelength. Subsequently, inversion is carried out for polydisperse SiO_2_@Au core–shell nanoparticles, in which four representative core size–shell thickness combination systems are configured to systematically analyze the effects of three key pre-inversion parameters, namely the particle size step, the wavelength range, and the wavelength step on the ill-conditioning of the inversion matrix. Finally, random noise levels of 0.01%, 0.1%, 0.5%, and 1% are introduced into the extinction spectra to investigate the robustness of the proposed method.

### 3.1. Feasibility Analysis of Two-Dimensional Inversion

The light extinction properties of SiO_2_@Au core–shell nanospheres are co-regulated by the core radius *R_c_* and shell thickness *t*. Establishing a one-to-one mapping relationship between these two geometric parameters and the resonance features of the extinction spectrum is the fundamental prerequisite for achieving simultaneous inversion of the two-dimensional particle size distribution in terms of both core radius and shell thickness. In this section, based on Mie scattering theory for core–shell structures, the modulation rules of the core radius and shell thickness of monodisperse SiO_2_@Au nanospheres on the normalized extinction spectrum are systematically analyzed. The unique correspondence between the dual geometric parameters and the extinction spectral features is verified, thereby providing a theoretical basis for the subsequent inversion parameter selection.

**Figure 3:**
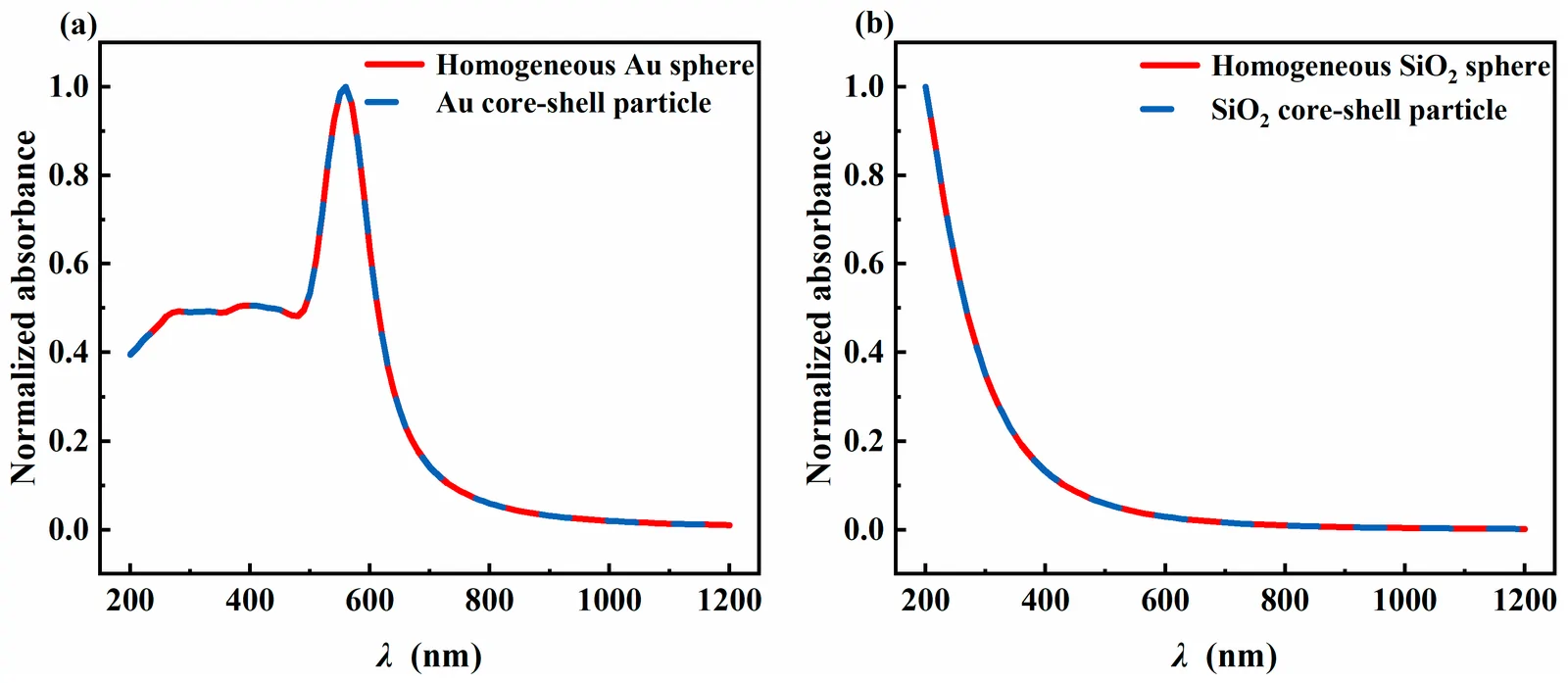
Validation of the core–shell model using homogeneous-sphere limiting cases with an identical outer radius of 44 nm. (**a**) Comparison between a homogeneous Au sphere and a core–shell particle with zero SiO_2_-core diameter; (**b**) comparison between a homogeneous SiO_2_ sphere and a core–shell particle with zero Au-shell thickness. The particle concentration, surrounding medium refractive index, wavelength range, and normalization procedure were kept identical in each comparison.

Before applying the core–shell model to particles with different core and shell properties, we verified its consistency with the conventional homogeneous-sphere Mie solution using two limiting cases. First, the Au-shell thickness was set to zero, reducing the core–shell particle to a homogeneous SiO_2_ sphere. The calculated absorbance spectrum was compared with that of a homogeneous SiO_2_ sphere having the same diameter. Second, the SiO_2_-core diameter was set to zero, reducing the particle to a homogeneous Au sphere. The resulting spectrum was compared with that of a homogeneous Au sphere with the same diameter, using the complex refractive index of Au. In both comparisons, the particle concentration, surrounding medium, wavelength range, and normalization procedure were kept identical. As shown in Figure 3, the normalized extinction spectra calculated using the core–shell formulation agree closely with those obtained from the conventional Mie solution for both SiO_2_ and Au spheres. These results confirm that the core–shell calculation reproduces the homogeneous-sphere limit for both dielectric and metallic particles. Having established this consistency, we next considered particles with independently varying core radii and shell thicknesses, for which the core and shell have different optical properties, and the particle population is described by prescribed distributions of the core radius and shell thickness.

**Figure 4:**
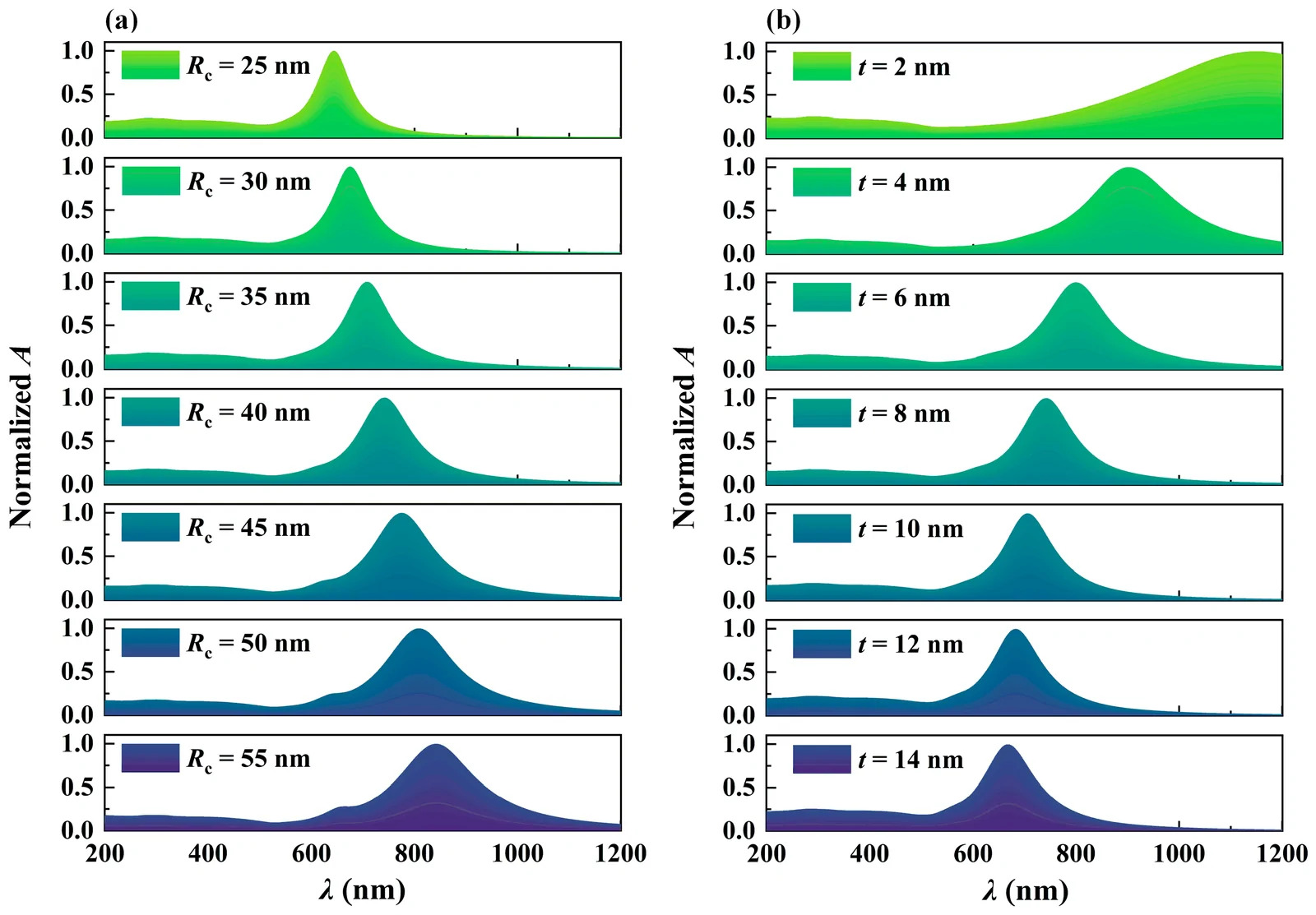
Regulation of normalized extinction spectra by two-dimensional particle size of monodisperse SiO_2_@Au core–shell nanospheres. (**a**) Shell thickness is 8 nm; (**b**) core radius is 40 nm.

With the shell thickness fixed at 8 nm, the core radius is varied from 25 nm to 55 nm in increments of 5 nm, as shown in Figure 4a. As the core radius increases, the resonance peak exhibits a pronounced redshift. This redshift originates from the increase in core radius, which primarily alters the overall curvature of the shell layer, thereby weakening the restoring force of the surface charges on the conduction electrons and reducing the confinement of the collective electron oscillations, ultimately leading to a decrease in resonance energy. Similarly, with the core radius fixed at 40 nm, the shell thickness is increased from 2 nm to 14 nm in increments of 2 nm, as shown in Figure 4b. As the shell thickness decreases, the primary resonance peak of the normalized extinction spectrum undergoes a dramatic redshift, monotonically evolving toward longer wavelengths.

It can thus be concluded that the modulation mechanisms of *t* and *R_c_* on the optical properties of SiO_2_@Au nanospheres are completely independent. The shell thickness directly governs the electromagnetic coupling strength by altering the spacing between the inner and outer surfaces, whereas the core radius dominates the charge restoring force by modifying the geometric curvature and radiation damping. Consequently, the two parameters exhibit significant differences in their dominant spectral bands and spectral evolution gradients with respect to the extinction features. The combination of these two parameters exhibits practical numerical identifiability from the broadband extinction spectral characteristics under the tested conditions, thereby physically excluding the degeneracy problem in which different (*R_c_*, *t*) combinations would yield identical extinction spectra. This result conclusively demonstrates that the simultaneous retrieval of the two-dimensional particle size distribution in terms of both the core radius and shell thickness of SiO_2_@Au core–shell nanospheres via extinction spectral inversion is theoretically well-founded and feasible. The structure–property relationship between the dual parameters and the extinction features established in this section provides theoretical support for the subsequent optimization of the particle size discretization step and wavelength-related parameters in the inversion process.

This design was selected for both physical and methodological reasons. From the physical perspective, the core radius determines the overall particle scale and affects the electromagnetic interaction associated with the dielectric core, whereas the shell thickness changes the relative contribution of the metallic shell and modifies the core–shell geometrical ratio. Variations in these two parameters can consequently alter the spectral position, amplitude, bandwidth, and sensitivity of the extinction response. Considering the two parameters at two representative levels allows the effects of core size, shell thickness, and their combined variation to be examined in a structured manner. From the methodological perspective, the four configurations provide four different optical and inverse-problem regimes. The two diagonal configurations, namely small-core/thin-shell and large-core/thick-shell, represent relatively distinct structural scales and core–shell geometries. The two mixed configurations, namely small-core/thick-shell and large-core/thin-shell, are important for examining cases in which the effects of the core radius and shell thickness vary in different directions. This arrangement helps assess whether the inversion performance and the suitable numerical parameters are controlled primarily by the overall particle size, the shell thickness, or the combined core–shell geometry. Therefore, the four configurations were not selected simply to increase the number of numerical examples. They were designed to provide a controlled sampling of the investigated two-dimensional structural parameter space and to examine the structure dependence of the extinction spectrum, the inverse kernel, and the reconstruction performance. These configurations are representative test cases within the parameter ranges considered in this work, rather than an exhaustive classification of all possible SiO_2_@Au core–shell nanoparticle structures.

**Figure 5:**
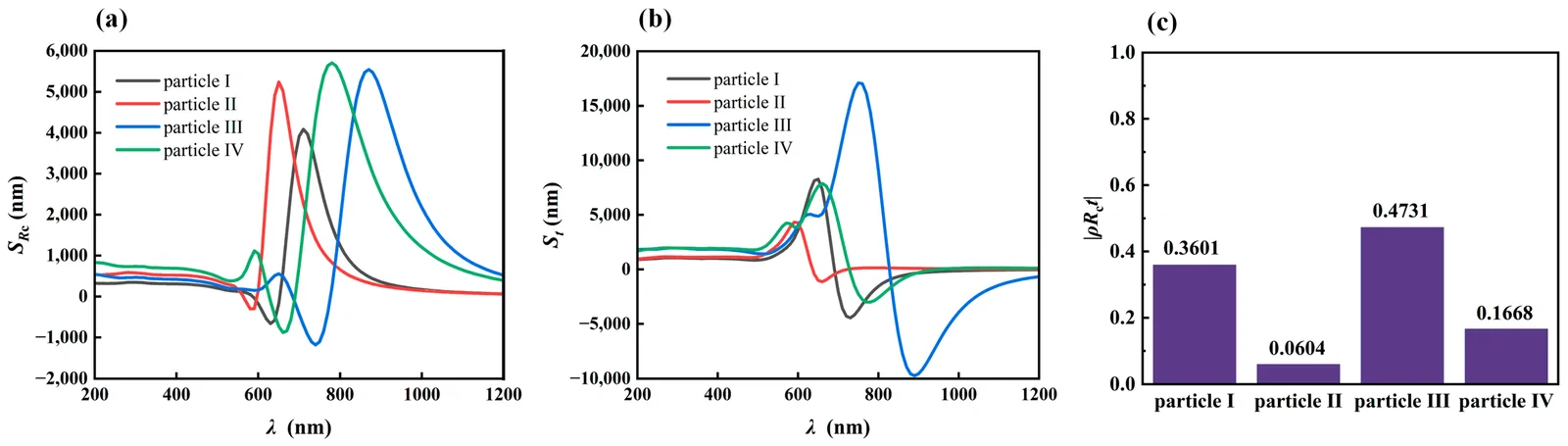
Wavelength-dependent local sensitivities of the extinction cross-section to (**a**) the core radius, *S_Rc_*, and (**b**) the shell thickness, St, for four representative structures. (**c**) Absolute correlation coefficients between the two sensitivity spectra for particles I–IV.

Figure 5a–c provide quantitative evidence for the distinguishability of the core radius and shell thickness from the extinction spectra. Figure 5a,b show that the four representative structures exhibit substantially different wavelength-dependent sensitivities to the core radius and shell thickness, respectively. The sensitivity curves differ in peak intensity, peak position, zero-crossing wavelength, and negative-response region, indicating that variations in these two structural parameters generate different spectral signatures rather than merely changing the extinction magnitude in the same manner. The differences are particularly evident in the long-wavelength region, where the four structures show distinct signs and amplitudes of the sensitivity responses.

The parameter coupling was further evaluated using the absolute correlation between the two sensitivity spectra, as shown in Figure 5c. The corresponding values for particles I–IV are 0.3601, 0.0604, 0.4731, and 0.1668, respectively. Particle II exhibits the weakest correlation, indicating that the spectral responses to the core radius and shell thickness are nearly independent for this structure and that the two parameters can be most readily distinguished. Particle IV also shows weak parameter coupling, whereas particle III has the largest correlation and consequently the strongest local similarity between the two parameter-induced responses. These results demonstrate that the ability to distinguish the two structural parameters depends on the selected particle structure and that particles II and IV provide more favorable conditions for parameter separation.

The representative unimodal distributions were selected as benchmark cases for evaluating the inversion method. The proposed method does not require the particle size distribution to follow a prescribed parametric form; it reconstructs the distribution directly on the discretized particle size grid subject to the non-negativity constraint. Thus, the use of unimodal distributions in the present tests reflects the choice of representative benchmark cases rather than a restriction of the method to unimodal distributions.

### 3.2. Effect of Particle Size Step

In the inversion process, the core radius step size (Δ*R_c_*) and the shell thickness step size (Δ*t*) directly determine the characteristic resolution of the inversion grid and the degree of ill-conditioning of the inversion equation system, making them the critical pre-inversion parameters for two-dimensional particle size distribution retrieval. An excessively large step size tends to obscure fine details of the particle size distribution, whereas an excessively small step size aggravates the ill-conditioning, leading to heightened sensitivity of the regularization parameter, intensified oscillation of the numerical solution, and amplified inversion errors. To quantify the coupled influence of step size on inversion accuracy, a full-factorial combination matrix of Δ*R_c_* and Δ*t* is constructed for four representative polydisperse SiO_2_@Au core–shell nanoparticle systems in this section. The influence of step size on inversion accuracy is systematically quantified, and the optimal particle size step size combination corresponding to each nanoparticle system is identified, providing standardized pre-inversion parameters for subsequent inversion optimization.

Numerical tests are performed on the inversion algorithm to evaluate its accuracy in retrieving particle size distributions from extinction spectra. In the numerical tests, the particle size distribution function is assumed to follow a two-dimensional Gaussian form, and the resulting particle size distribution function of the polydisperse SiO_2_@Au core–shell nanoparticle system is expressed as

(26) $$f(R_{c},t)=\frac{1}{2\pi \sigma_{\mathrm{Rc}}\sigma_{t}}\exp\left(-\frac{{\left(R_{c}-\mu_{\mathrm{Rc}}\right)}^{2}}{2\sigma_{\mathrm{Rc}}^{2}}-\frac{{\left(t-\mu_{t}\right)}^{2}}{2\sigma_{t}^{2}}\right)$$

where *μ_Rc_* is the mean core radius of the particle system, *σ_Rc_* is the standard deviation of the core radius, *μ_t_* is the mean shell thickness of the particle system, and *σ_t_* is the standard deviation of the shell thickness. Accordingly, the reference particle size parameters of the polydisperse SiO_2_@Au core–shell nanoparticle system should be prescribed in advance.

Four representative polydisperse SiO_2_@Au core–shell nanoparticle systems are configured, all of which follow normal distributions. Through extensive data analysis, it is found that the inversion relative error is closely correlated with both the original and inversion step sizes of the particle size distribution. Accordingly, four representative nanoparticle systems covering small-core/large-core and thin-shell/thick-shell configurations are selected under varying step sizes of core radius and shell thickness, with the specific parameters listed in Table 1. The core radius step size is set at five levels, 0.2 nm, 0.4 nm, 0.6 nm, 0.8 nm, and 1.0 nm, while the shell thickness step size is set at five levels, 0.1 nm, 0.2 nm, 0.3 nm, 0.4 nm, and 0.5 nm. These combinations form a 5 × 5 full-factorial matrix, yielding a total of 25 step size parameter combinations. The inversion relative error is adopted as the evaluation metric.

Inversion calculations were performed for 25 combinations of discretization step sizes, and the relative inversion errors of the parameters for the four particle systems were obtained. The optimal step size combination for each particle system is presented in Table 2. The last column lists, in sequence, the relative inversion errors of the mean core radius, standard deviation of the core radius, mean shell thickness, standard deviation of the shell thickness, and particle number concentration for the polydisperse SiO_2_@Au core–shell nanoparticle systems. The results show that the optimal step size combinations are Δ*R_c_* = 0.8 nm and Δ*t* = 0.2 nm for particle system I, Δ*R_c_* = 0.2 nm and Δ*t* = 0.5 nm for particle system II, Δ*R_c_* = 0.2 nm and Δ*t* = 0.1 nm for particle system III, and Δ*R_c_* = 0.4 nm and Δ*t* = 0.1 nm for particle system IV. Significant differences are observed in the optimal step sizes among particle systems with different structural characteristics: large-core systems generally require smaller Δ*R_c_* values than small-core systems, while thin-shell systems generally require smaller Δ*t* values than thick-shell systems.

The differences in optimal step sizes among nanoparticle systems with distinct geometrical configurations fundamentally originate from the varying modulation sensitivities of the core–shell geometric parameters to the extinction spectral features. On one hand, the electron oscillation modes of the Au shell and the plasmon hybridization intensity directly govern the sensitivity of the extinction spectrum to variations in the shell thickness step size. In thin-shell structures with *t* = 8 nm, the Au shell resides in a transitional regime between quantum confinement effects and bulk-like behavior, where the coupling between the inner and outer surface plasmons is exceptionally strong. In this regime, a subtle variation of 0.1 nm in shell thickness is sufficient to induce a shift of 2–3 nm in the LSPR peak position.

On one hand, for the large-core thin-shell structure, the lower shell curvature results in weaker geometric confinement of electron oscillations, which further enhances the sensitivity to thickness variations; consequently, the optimal Δ*t* decreases to 0.1 nm. In contrast, the small-core thin-shell structure, with its higher curvature, exhibits a relatively gradual variation in coupling effects, yielding an optimal Δ*t* of 0.2 nm. For thick-shell structures with *t* = 14 nm, the Au shell is dominated by bulk-like properties, the coupling between the inner and outer surfaces is significantly weakened, and the sensitivity of the extinction spectrum to thickness variations is correspondingly reduced. For the large-core thick-shell structure, although the overall sensitivity decreases, the relatively low curvature still maintains a comparatively high sensitivity to thickness variations, yielding an optimal Δ*t* of 0.1 nm. In contrast, the small-core thick-shell structure, owing to its extremely high curvature, exhibits minimal influence of thickness variations on the coupling effects; consequently, a larger step size of 0.5 nm is sufficient to meet the accuracy requirements, while also effectively preventing the aggravation of ill-conditioning in the inversion equation system. On the other hand, the core radius indirectly modulates the LSPR by tuning the overall curvature of the Au shell. For the small-core thin-shell structure, the shell thickness is moderate, and a variation of 0.8 nm in the core radius is sufficient to induce a noticeable shift in the LSPR peak position. Adopting a step size of 0.8 nm enables complete capture of the variations in extinction features while avoiding excessive inversion dimensionality that would aggravate ill-conditioning, thereby achieving an optimal balance between accuracy and stability. In contrast, for thick-shell and large-core structures, the reduced shell curvature significantly enhances the modulation effect of subtle core radius variations on the LSPR. Therefore, smaller step sizes of 0.2–0.4 nm are required to precisely distinguish the differences in extinction spectra corresponding to different core radii.

It should be noted that this distribution is assumed solely for the purpose of numerical testing of the inversion problem. In practice, when employing the extinction spectroscopy method, prior knowledge of the particle size distribution function is not required, and the particle size distribution can take any arbitrary form. For structural parameters with high sensitivity, a smaller step size should be adopted to ensure adequate feature resolution; for structural parameters with low sensitivity, a larger step size can be employed to mitigate the ill-conditioning of the inversion problem.

The optimized wavelength ranges, wavelength intervals, and particle size discretization steps obtained in these tests should be interpreted as configuration-specific recommendations under the optical model, parameter ranges, noise conditions, and numerical settings considered in this study. They should not be regarded as universal parameter values that can be directly transferred to arbitrary SiO_2_@Au core–shell nanoparticle systems. The generalizable result of the present analysis is the structure-dependent parameter-selection procedure. For a new particle configuration, the same procedure can be applied by recalculating the extinction kernel over the corresponding core radius and shell thickness ranges, constructing candidate wavelength and discretization schemes, and evaluating the resulting inversion performance using the same accuracy and stability criteria adopted in this study. The mean and standard deviation errors of the retrieved core radius, shell thickness, and concentration can then be used to identify a suitable parameter combination for the new configuration. Thus, the transferable aspect of this study is the relationship between the particle configuration and the parameter-optimization procedure rather than the direct universal transfer of the specific numerical values obtained for the four configurations.

### 3.3. Effect of Wavelength Range

The wavelength range of the extinction spectrum directly determines the amount of optical characteristic information contained in the inversion equation system, and thus serves as a critical parameter governing the accuracy of two-dimensional particle size distribution retrieval. Based on the optimal particle size step combinations identified for each nanoparticle system in the preceding section, the influence of different wavelength ranges on inversion accuracy is systematically investigated, and the optimal wavelength range corresponding to each structurally distinct nanoparticle system is determined, thereby further improving the accuracy and practical applicability of the inversion method.

Four representative wavelength intervals are configured within the 200–1200 nm spectral range, covering the commonly used spectral acquisition ranges in experimental practice: 200–1200 nm (full band); 200–400 nm (ultraviolet band); 400–700 nm (visible band); 700–1200 nm (near-infrared band). The original and retrieved two-dimensional particle size distributions under different wavelength ranges are presented in Figure 4. Surprisingly, for nanoparticle system I, the optimal inversion result is achieved by employing solely the ultraviolet narrow band of 200–400 nm; whereas for the remaining three configurations (nanoparticle systems II–IV), the full-band broadband features spanning 200–1200 nm are indispensable for attaining the best inversion performance.

**Figure 6:**
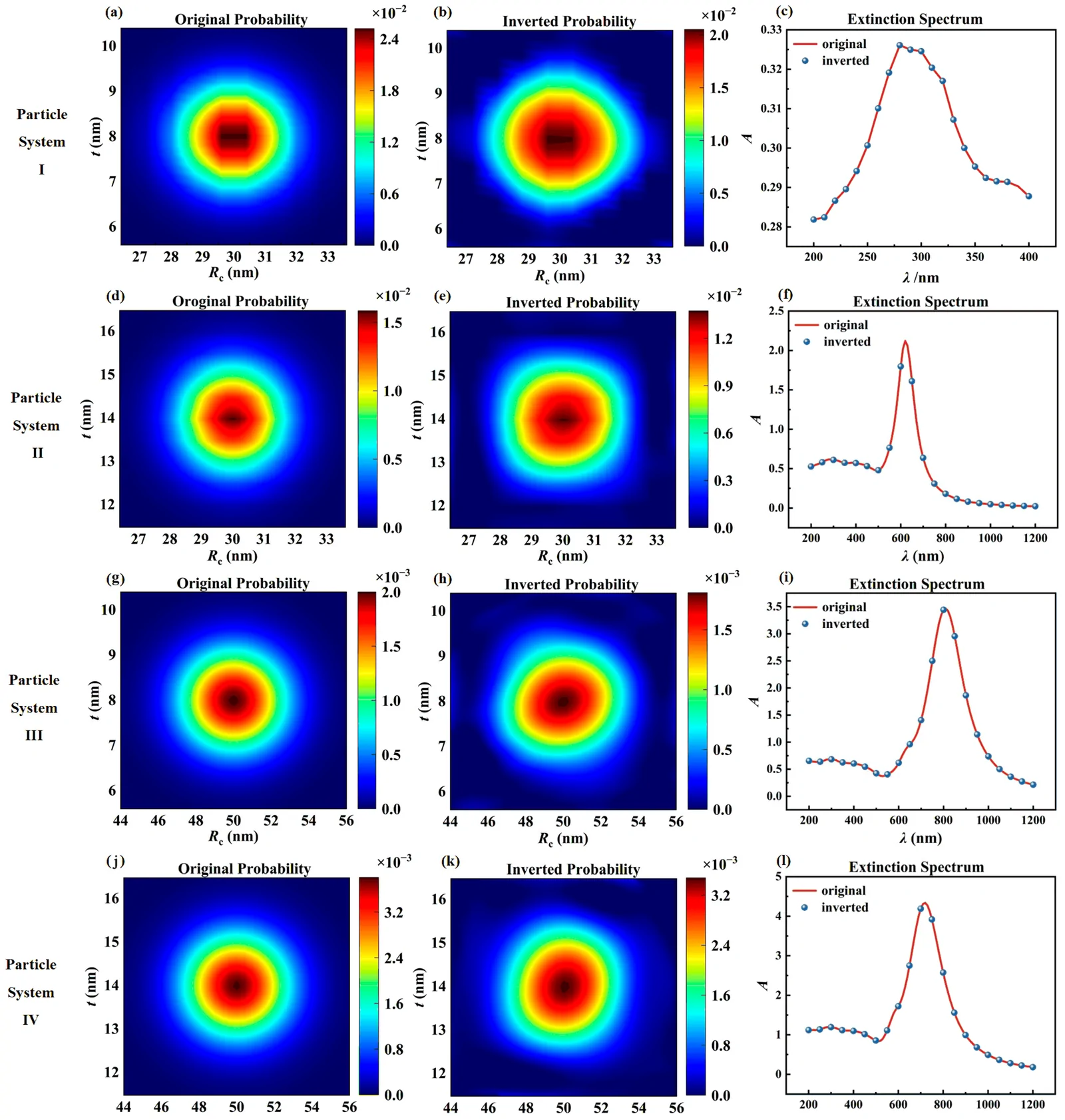
Inversion results of four typical SiO_2_@Au core–shell nanoparticle systems under the optimal wavelength range. (**a**–**c**) Particle system I (200–400 nm); (**d**–**f**) particle system II (200–1200 nm); (**g**–**i**) particle system III (200–1200 nm); (**j**–**l**) particle system IV (200–1200 nm).

Figure 6 summarizes the inversion results and extinction spectra for four representative SiO_2_@Au core–shell nanoparticle systems. For small-core thin-shell structures with extremely small overall dimensions, the electromagnetic behavior approaches the quasi-static limit. Within this size regime, the polarization response at long wavelengths decays rapidly, leaving essentially only a smooth long-wavelength tail governed by Rayleigh scattering. In this specific configuration, as shown in Figure 6a–c, forcibly incorporating the long-wavelength band of 400–1200 nm into the matrix computation is tantamount to injecting a large volume of highly linearly correlated and informationally redundant smooth data into the equation system. This not only fails to provide new orthogonal information for discriminating the two structural parameters, but further dilutes the spectral weight of the ultraviolet region, ultimately aggravating the ill-conditioning of the inversion matrix and significantly degrading the stability of the solution. Therefore, for small-core thin-shell structures, truncating the long-wavelength band and retaining only the high-frequency ultraviolet data represents the optimal strategy for eliminating algebraic redundancy and extracting effective characteristic information. Once the size regime extends beyond the small-size domain, the phase retardation of the internal electromagnetic field and the radiation damping increase exponentially. Within this size range, the originally strongly coupled optical modes progressively decouple. The higher-order plasmonic modes remain concentrated in the ultraviolet band (200–400 nm), whereas the dominant dipolar resonance peak undergoes an exceptionally pronounced redshift, shifting entirely into the visible or even near-infrared band (600–1100 nm). As illustrated in Figure 6d–l, breaking this spectral mimicry cannot rely solely on a single wavelength band. Truncation at any single wavelength range will inevitably result in the loss of critical inversion features. If only the ultraviolet band is employed for inversion, key optical characteristics such as the LSPR primary peak position and linewidth are lost, causing the inversion model to exhibit multiple solutions due to insufficient constraints. Conversely, if only the near-infrared band is utilized, the spectral signal degenerates into a resonance long-wavelength tail devoid of characteristic gradients, rendering the parameter localization entirely without a reliable basis for discrimination. Therefore, for the three configurations comprising small-core thick-shell, large-core thin-shell, and large-core thick-shell structures, incorporating the full-spectrum range of 200–1200 nm constitutes a necessary prerequisite for achieving parameter decoupling.

It can be concluded that the optimal inversion wavelength range is not a universally applicable fixed constant, but rather represents a dynamic balance between the effective extraction of spectral characteristic information and the suppression of ill-conditioning in the inversion matrix. The rationale for truncating the long-wavelength band in small-core thin-shell structures is to eliminate the redundant smooth and featureless data in the long-wavelength region, thereby preventing the high linear correlation among matrix column vectors from causing solution divergence. In contrast, for large-core and thick-shell systems, the adoption of full-spectrum acquisition serves to comprehensively capture the phase retardation effects that are substantially enhanced with increasing spatial scale, thereby constructing sufficiently orthogonal physical constraints across an exceptionally broad spectral span to satisfy the stringent requirements of dual-parameter decoupling.

### 3.4. Effect of Wavelength Step Size

The wavelength discretization step size determines the sampling resolution of the extinction spectrum, directly governing the dimensionality, computational efficiency, and solution accuracy of the inversion equation system, and thus constitutes a critical parameter that must be explicitly determined for the experimental implementation of the inversion method. Based on the optimal particle size step combinations and optimal wavelength ranges identified through the preceding sequential screening procedure, this section systematically evaluates the influence of three representative sampling resolutions (Δ*λ* = 10 nm, 5 nm, 2 nm) on inversion accuracy, establishes the optimization selection principles for wavelength step size, and achieves a balanced trade-off between inversion accuracy and computational efficiency.

The wavelength step size yielding the minimum inversion error for each nanoparticle system is summarized in Table 3. The results demonstrate that, with the exception of nanoparticle system I, the inversion accuracy of the remaining three systems improves monotonically with increasing wavelength step size. At a step size of Δ*λ* = 10 nm, the relative errors of both the mean core radius and shell thickness remain below 10^−3^%, while the number concentration error falls below 10^−5^%, with the retrieved distributions exhibiting near-perfect agreement with the original profiles. For nanoparticle system I, the optimal wavelength step size is determined to be 5 nm. This is attributed to the relatively narrow LSPR peak of this configuration, where a 10 nm step size leads to the loss of fine spectral variations in the peak profile, whereas a 5 nm step size enables complete capture of the characteristic features without introducing excessive redundant data.

The inversion accuracy does not improve monotonically with decreasing wavelength step size. This counterintuitive phenomenon is fundamentally attributed to the coupling effect between the characteristic redundancy of the extinction spectrum and the ill-conditioning of the inversion equation system, which can be elucidated from two complementary perspectives. The first concerns the suppression of ill-conditioning in the inversion equation system. An increase in wavelength step size implies a reduction in the number of sampling points, thereby lowering the dimensionality of the inversion equation system. Consequently, the linear correlation among the column vectors of the extinction cross-section matrix is substantially weakened, effectively mitigating the inherent ill-conditioning of the inversion problem. The non-negative Tikhonov regularization method exhibits higher solution stability and weaker noise amplification for low-dimensional, low-ill-conditioned equation systems, and consequently the inversion error is reduced rather than increased. For nanoparticle systems II-IV, the LSPR peaks are comparatively broad, and a sampling density of Δ*λ* = 10 nm is already sufficient to completely preserve all core spectral characteristics, including the resonance peak position, peak profile, and extinction intensity. Further reduction in the step size only introduces additional highly correlated sampling points, exacerbating the linear dependence among matrix column vectors, causing a sharp increase in the condition number, deteriorating the ill-conditioning, and ultimately elevating the inversion error. The second mechanism concerns the filtering effect of extinction characteristic redundancy. In a continuous extinction spectrum, the extinction cross-section data at adjacent wavelength points exhibit extremely high mutual correlation, particularly in the smooth regions flanking both sides of the resonance peak. The fine sampling at Δ*λ* = 2 nm introduces a substantial amount of redundant spectral information, which not only fails to enhance the inversion accuracy but instead causes the condition number of the inversion equation system to grow exponentially, resulting in the amplification of minor numerical errors by the ill-conditioned matrix and ultimately manifesting as a degradation in inversion accuracy. For nanoparticle system I, the LSPR peak is comparatively narrow with more intricate peak profile variations, such that a 10 nm step size leads to the loss of fine structural details in the peak profile, causing a marginal increase in the inversion error. In contrast, a 5 nm step size achieves the optimal balance between accuracy and stability by completely capturing the fine characteristic features of the peak profile while simultaneously filtering out the majority of redundant spectral data.

It can be concluded that the wavelength step size exerts a significant influence on the inversion accuracy of the two-dimensional particle size distribution of SiO_2_@Au core–shell nanospheres. The inversion accuracy does not improve monotonically with decreasing step size, and an optimal wavelength step size interval exists. The selection of the optimal wavelength step size should be made in conjunction with the LSPR peak width of the core–shell structure: narrow-peak configurations require a smaller step size to preserve fine spectral features, whereas broad-peak configurations can accommodate a larger step size to achieve a favorable balance between inversion accuracy and computational efficiency.

### 3.5. Effect of Random Noise

In practical measurements, measurement errors constitute an inevitable influencing factor. The acquisition of extinction spectra invariably introduces random noise, which distorts the characteristic features of the extinction spectrum and compromises the inversion accuracy. Consequently, the noise robustness of the inversion method serves as a core evaluation criterion for its practical applicability. Based on the optimal particle size step, wavelength range, and wavelength step size obtained from the preceding full-parameter optimization, this section examines the robustness of the inversion framework by introducing random noise levels of 0.01%, 0.1%, 0.5%, and 1% into the original extinction spectra. This range represents progressively increasing measurement uncertainty, from weak to relatively strong perturbations, and is used to evaluate the effects of noise on the reconstructed two-dimensional particle size distribution and number concentration, as well as the practical applicability of the proposed method.

**Figure 7:**
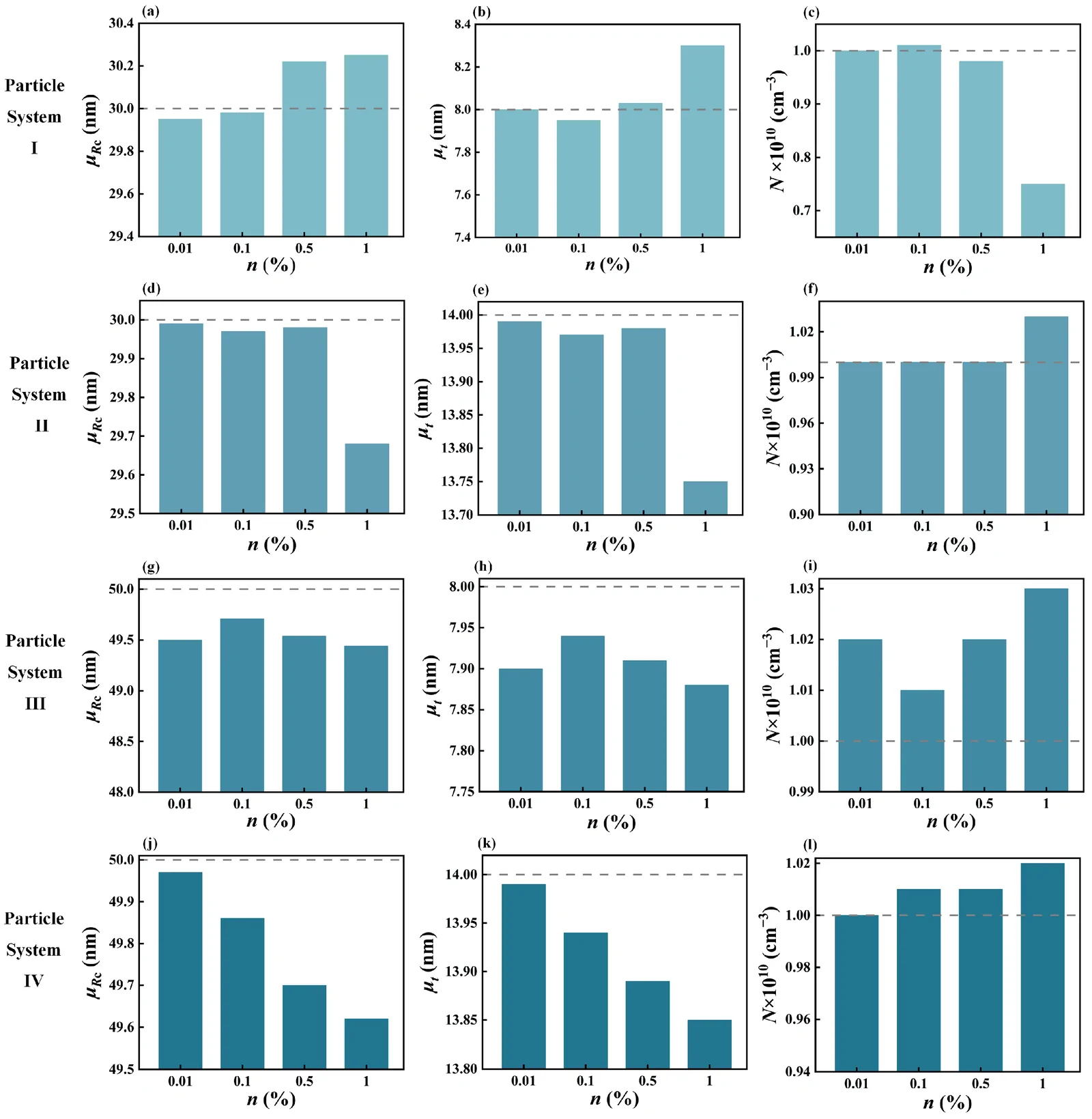
Inversion results of four typical SiO_2_@Au core–shell nanoparticle systems under different random noise levels. (**a**–**c**) Particle system I; (**d**–**f**) particle system II; (**g**–**i**) particle system III; (**j**–**l**) particle system IV.

The inversion results for the core radius, shell thickness, and number concentration of the four particle systems with varying random noise levels are presented in Figure 7. In each subplot, the dashed horizontal line represents the corresponding mean value of the particle-structure parameter for the respective particle system and serves as the reference value for evaluating the inversion results. The results demonstrate that the core mean parameters exhibit excellent noise robustness. Within the experimentally relevant random noise range of 0.01–1%, the relative inversion errors of the mean core radius and mean shell thickness for all four nanoparticle systems remain below 2%. Among them, the large-core thick-shell system delivers the most outstanding performance: under high-intensity noise of 1%, the relative error of the mean core radius is merely 0.757% and that of the mean shell thickness is only 1.060%, fully satisfying the accuracy requirements of practical experimental measurements. Regarding the particle number concentration, the influence of noise exhibits a clear dependence on the particle configuration. For the large-core configurations, the concentration inversion error remains below 3% under a noise level of 1%. In contrast, the error increases substantially for the small-core configurations, reaching 24.56% for the small-core/thin-shell system. This result does not indicate that all inversion parameters are equally sensitive to noise. Rather, it reflects the different types of spectral information used to retrieve the particle concentration and the structural parameters.

In contrast, the mean core radius and shell thickness are more strongly associated with spectral-shape information, including the relative variation in the spectrum with wavelength, the position of characteristic spectral features, and the change in spectral curvature. For the small-core/thin-shell system, the extinction cross-section of an individual particle is relatively small, and the corresponding extinction response contains weaker concentration-related amplitude information than that of the larger-core or thicker-shell configurations. As a result, the concentration-related direction in the inverse problem is less strongly distinguished from the noise-perturbed spectral data. Small perturbations in the extinction spectrum can therefore produce a comparatively large change in the recovered concentration, even when the main spectral-shape information remains sufficiently stable for retrieving the mean core radius and shell thickness. The different error levels can also be understood from the conditioning of the inverse problem. The concentration is reconstructed from the cumulative contribution of the particle size and shell thickness distribution to the extinction spectrum. When the useful extinction response is weak, the concentration-related component of the inversion matrix can have a lower effective sensitivity and can be more strongly affected by correlations with neighboring size and shell thickness components. This amplifies the uncertainty in the recovered concentration. By comparison, the mean core radius and shell thickness are calculated from the reconstructed distribution and are constrained by the wavelength-dependent shape of the response. The corresponding spectral-shape information can remain identifiable even when the absolute amplitude is perturbed, which explains why their relative errors remain below 2% for the small-core system under the same 1% noise level. Therefore, the 24.56% error should be interpreted as a structure-dependent limitation of concentration retrieval under weak extinction signals, rather than as a failure to retrieve the mean structural parameters. These results indicate that the proposed method retains good robustness for estimating the mean core radius and shell thickness, whereas the quantitative accuracy of concentration retrieval is reduced for the small-core/thin-shell configuration under 1% noise.

From an algorithmic perspective, the inherent noise suppression capability of non-negative Tikhonov regularization is the core reason for the high stability of the proposed method. The core of this method lies in incorporating non-negativity constraints and a regularization penalty term into the least-squares fitting objective function

min{‖**Ax** − **b**‖^2^ + *α*‖**Lx**‖^2^} (27)

which sacrifices partial fitting accuracy in exchange for solution stability. In essence, this approach applies damping factors to the small singular values of the kernel matrix, directly suppressing the amplification of high-frequency noise components and effectively preventing random noise from being infinitely amplified by the ill-conditioned inversion matrix, thereby achieving inherent noise suppression at the algorithmic level. The mean parameters also benefit from inherent statistical robustness. As the first-order moments of the particle size distribution, the mean core radius and mean shell thickness are governed by the overall characteristics of the extinction spectrum. Because the superimposed Gaussian white noise is zero-mean, its positive and negative contributions offset each other when integrated across the full wavelength range, resulting in a negligible effect on the inverted distribution means. The particle number concentration, which is likewise determined by the total integrated extinction intensity, exhibits similarly strong robustness. In addition, by jointly optimizing the particle size step, wavelength range, and wavelength step size, the ill-conditioning of the inversion system is reduced to a minimum, further strengthening the noise robustness of the algorithm. As a result, the inversion accuracy of the key parameters remains acceptable even at a noise level as high as 1%.

**Figure 8:**
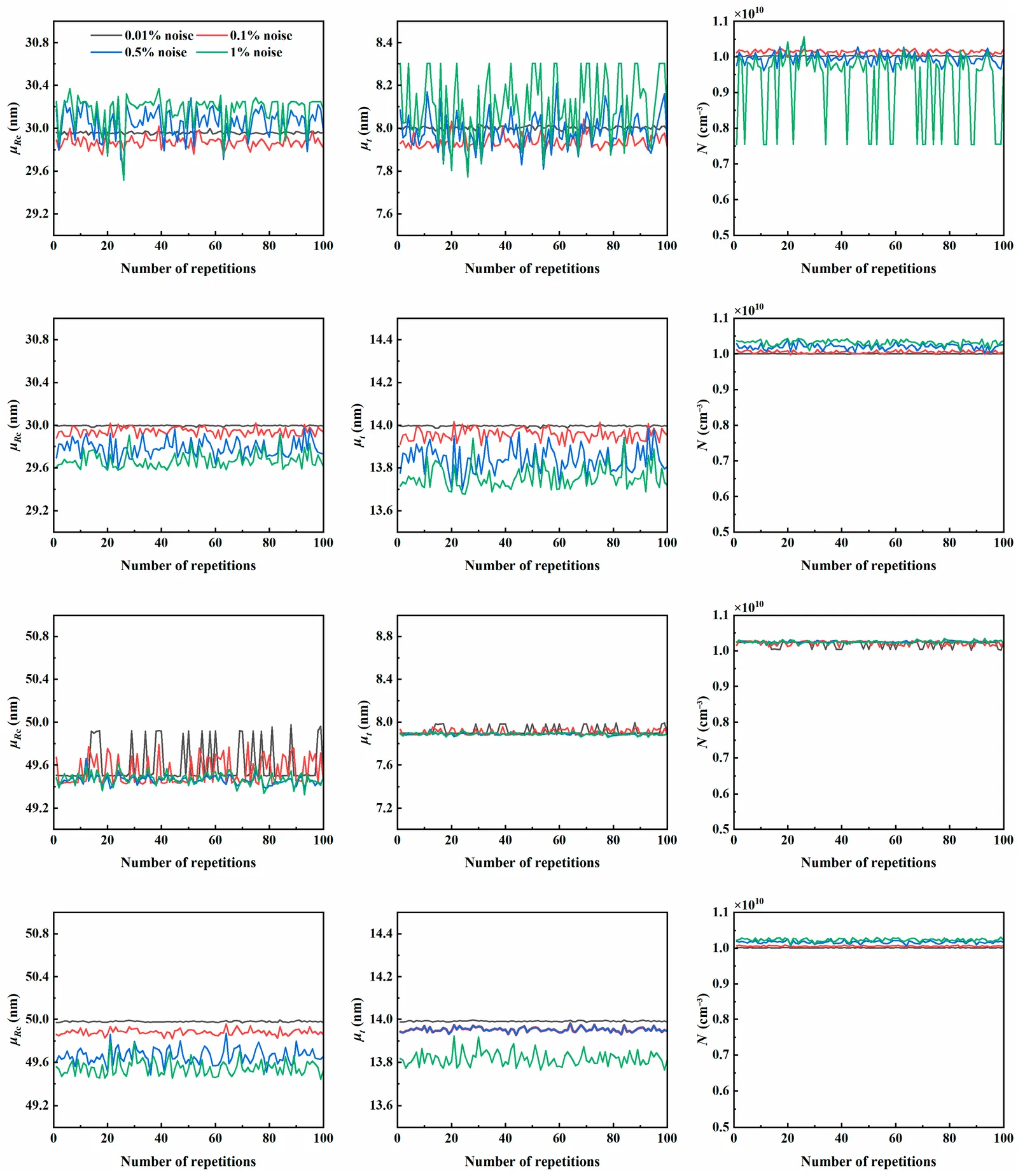
Statistical distributions of the retrieved mean core radius, mean shell thickness, and particle number concentration for four representative SiO_2_@Au core–shell nanoparticle structures under four random noise levels.

The results presented above were obtained from a single random noise realization at each noise level. To further evaluate the statistical reproducibility and robustness of the inversion method, multiple independent noise realizations were subsequently performed. Four representative SiO_2_@Au core–shell nanoparticle structures and four random noise levels were considered. For each combination of particle structure and noise level, 100 independent noise realizations were generated and added to the corresponding extinction spectrum. The inversion was then performed separately for each realization using the same optimized numerical parameters. Thus, a total of 1600 independent inversion tests were conducted. For each test, the mean core radius, mean shell thickness, and particle number concentration were calculated from the retrieved two-dimensional size distribution. The statistical results of these three parameters over the 100 independent realizations for each structure–noise combination are presented in Figure 8.

Figure 8 provides a statistical evaluation of the inversion performance over multiple independent noise realizations. For each of the four particle structures, the retrieved mean core radius, mean shell thickness, and particle number concentration were obtained from 100 independently perturbed extinction spectra at each noise level. The spread of the retrieved values therefore reflects the sensitivity of the inversion results to the specific realization of the random noise, rather than the effect of a single noise sequence. The results show that the retrieved mean core radius and mean shell thickness remain relatively stable across the 100 independent realizations, even as the noise level increases. Overall, the 100 independent realizations for each structure–noise combination demonstrate that the observed inversion performance is statistically reproducible. The results also provide a more comprehensive assessment of the robustness of the proposed method than a single noise realization, supporting its potential for the nondestructive and high-throughput characterization of SiO_2_@Au core–shell nanoparticles.

The excellent robustness of the proposed two-dimensional size and concentration inversion method for SiO_2_@Au core–shell nanospheres thus arises from the combined contributions of the inherent noise suppression of non-negative Tikhonov regularization, the first-order moment statistical nature of the mean parameters, and the mitigation of inversion ill-conditioning through full-parameter optimization. Over the experimentally relevant noise range of 0.01–1%, the relative errors in the inverted mean core radius and mean shell thickness are consistently below 2%, confirming that the inversion framework operates effectively without requiring ideal noise-free conditions or demanding preprocessing. These results establish a robust foundation for high-throughput, non-destructive detection of SiO_2_@Au core–shell nanoparticles. The present numerical tests focus on random perturbations in the measured extinction intensity. Wavelength calibration errors and spectrally correlated instrumental errors represent different sources of uncertainty. Further evaluation involving baseline variations, wavelength calibration errors, and spectrally correlated noise would provide a more comprehensive assessment in future work.

## 4. Conclusions

In summary, a systematic parameter-configuration framework was established by integrating Mie scattering theory, extinction spectroscopy, and non-negative Tikhonov regularization. The effects of the core radius and shell thickness on the extinction spectrum in monodisperse systems were analyzed. The effects of the particle size step, wavelength range, and wavelength step size on the ill-posedness of the inversion matrix for four typical polydisperse systems were investigated, and standardized parameter combinations adapted to different structures were determined.

The independent modulation mechanisms of the core radius and shell thickness on the extinction spectrum were further investigated. The results indicate that these two parameters produce distinguishable spectral responses within the tested structural ranges and wavelength windows, although their effects remain partially coupled. Therefore, the proposed framework demonstrates practical numerical identifiability rather than a globally unique mapping between the structural parameters and the extinction spectrum. The results also show that the effects of the particle size step, wavelength range, and wavelength step size are strongly structure-dependent. The particle size step must balance spectral-feature resolution and inversion ill-conditioning, with thin-shell and large-core structures being more sensitive to variations in shell thickness and core radius, respectively. The optimal wavelength range for small-core/thin-shell structures is 200–400 nm in the ultraviolet region, whereas the other structures require the full 200–1200 nm range to decouple the structural parameters. Moreover, reducing the wavelength step size does not necessarily improve inversion accuracy: a 10 nm interval is sufficient for broad-peak structures, whereas a 5 nm interval is required for small-core/thin-shell structures with narrow peaks. Under the prescribed simulated noise levels of 0.01–1%, the errors in the key parameters remained below 2% for the tested systems, demonstrating the numerical robustness of the method under the specified conditions. This robustness arises from the combined effects of the optical characteristics of the core–shell structures and the regularized inversion scheme. The proposed framework provides a practical basis for the numerical assessment and potential quality control of core–shell nanoparticles and may be further extended to other composite nanostructures or combined with machine learning methods.

Several limitations should nevertheless be acknowledged. Further experimental validation involving wavelength calibration uncertainty, baseline drift, and spectrally correlated measurement errors would be valuable for assessing the performance of the method under more comprehensive experimental conditions. Because only one particle concentration was considered, the present work does not establish a general concentration limit for single-scattering approximation, and the applicability of the model to substantially higher concentrations requires additional validation. In addition, the present results are based on numerically generated extinction spectra. Experimental validation using independently characterized SiO_2_@Au nanoparticles is therefore required to assess the accuracy and applicability of the method to real samples.

## Figures and Tables

**Table 1:** Original particle size distribution parameters of four typical polydisperse SiO_2_@Au core–shell nanoparticle systems.

Particle Categories	*μ_Rc_* (nm)	σ*_Rc_* (nm)	*μ_t_* (nm)	*σ_t_* (nm)
Small core, thin shell (I)	30	1.2	8	0.8
Small core, thick shell (II)	30	1.2	14	0.84
Large core, thin shell (III)	50	2.0	8	0.8
Large core, thick shell (IV)	50	2.0	14	0.84

**Table 2:** Optimal particle size step combinations and corresponding minimum inversion relative errors for four typical nanoparticle systems.

Particle Categories	Δ*R_c_* (nm)	Δ*t* (nm)	*Eμ_Rc_*, *E*σ*_Rc_*, *Eμ_t_*, *Eσ_t_*, *E_N_* (%)
I	0.8	0.2	8.8682 × 10^−2^, 1.8022 × 10^1^, 1.9973 × 10^−2^, 1.2765 × 10^0^, 7.4645 × 10^−2^
II	0.2	0.5	5.9312 × 10^−6^, 1.2307 × 10^−3^, 1.9738 × 10^−5^, 1.0448 × 10^−3^, 9.3198 × 10^−6^
III	0.2	0.1	8.2509 × 10^−5^, 1.3649 × 10^−2^, 6.2776 × 10^−5^, 8.5714 × 10^−4^, 5.6206 × 10^−5^
IV	0.4	0.1	3.1482 × 10^−6^, 7.3010 × 10^−4^, 2.3435 × 10^−6^, 9.5728 × 10^−4^, 1.5422 × 10^−5^

**Table 3:** Inversion results and relative errors of the nanoparticle systems under the optimal wavelength step size.

Particle Categories	I	II	III	IV
Optimal Δ*λ* (nm)	5	10	10	10
*Eμ_Rc_* (%)	4.4768 × 10^−5^	5.9312 × 10^−6^	8.2509 × 10^−5^	3.1482 × 10^−6^
*E*σ*_Rc_* (%)	2.0986 × 10^−2^	1.2307 × 10^−3^	1.3649 × 10^−2^	7.3010 × 10^−4^
*Eμ_t_* (%)	1.5881 × 10^−5^	1.9738 × 10^−5^	6.2776 × 10^−5^	2.3435 × 10^−6^
*Eσ_t_* (%)	4.9440 × 10^−3^	1.0448 × 10^−3^	8.5714 × 10^−4^	9.5728 × 10^−4^
*EN* (%)	9.0209 × 10^−5^	9.3198 × 10^−6^	5.6206 × 10^−5^	1.5422 × 10^−5^

## Data Availability

The original contributions presented in this study are included in the article; further inquiries can be directed to the corresponding author.

## Abbreviations

The following abbreviations are used in this manuscript:

AFM	Atomic Force Microscopy
DLS	Dynamic Light Scattering
LSPR	Localized Surface Plasmon Resonance
